# Intraspecific differences in the invasion success of the Argentine ant *Linepithema humile* Mayr are associated with diet breadth

**DOI:** 10.1038/s41598-021-82464-1

**Published:** 2021-02-03

**Authors:** Yugo Seko, Koya Hashimoto, Keisuke Koba, Daisuke Hayasaka, Takuo Sawahata

**Affiliations:** 1 Graduate School of Agriculture, Kindai University, Nakamachi 3327-204, Nara, 631-8505 Japan; 2 Faculty of Agriculture, Kindai University, Nakamachi 3327-204, Nara, 631-8505 Japan; 3 Center for Ecological Research (CER), Kyoto University, Hirano 2-509-3, Otsu, Shiga 520-2113 Japan; 4 National Institute for Environmental Studies (NIES), Onogawa 16-2, Tsukuba, Ibaraki 305-8506 Japan

**Keywords:** Invasive species, Stable isotope analysis, Urban ecology, Invasive species, Stable isotope analysis, Urban ecology

## Abstract

The Argentine ant, *Linepithema humile* Mayr, has spread to almost all continents. In each introduced region, *L. humile* often forms a single large colony (supercolony), the members of which share the haplotype “LH1”, despite the presence of other supercolonies with different genetic structures. However, the mechanisms underlying the successful invasion of LH1 ants are unclear. Here, we examined whether diet breadth differs between more successful (LH1) and less successful (LH2, LH3, LH4) *L. humile* supercolonies in Japan to better understand the processes responsible for invasion success. The standard ellipse areas (SEAs) of *δ*^13^C and *δ*^15^N and their ranges (CR and NR) were used as diet breadth indices. The SEAs of LH1 were much larger than those of the less successful supercolonies despite no differences in the baseline SEAs of arthropods within the supercolony habitats, indicating that the invasion success of a supercolony is associated with its diet breadth. Furthermore, LH1 had a broader CR than the other supercolonies, suggesting that which might be derived from superior resource exploitation ability. Our study highlights the importance of focusing on intraspecific differences in diet breadth among supercolonies when assessing organisms that can potentially invade and become dominant in new habitats.

Biological invasions have increased with the increases in world tourism and trade. However, not all introduced species become successful invaders. To predict invasion success (i.e., successful post-invasion colonization), many researchers have attempted to identify the key traits of biological invaders that facilitate invasion success in new environments and habitats, such as aggressiveness^1,2^, thermal tolerance^3^, and diet breadth^4–6^. A typical approach for determining such key traits is to investigate differences between more- and less-successful populations of widespread, dominant species^7,8^. However, since studies employing this approach have often focused on interspecific differences in biological traits^3,5^, the roles of intraspecific differences in invasiveness and/or biological traits within conspecifics with different genotypes have been largely overlooked. Given that whether the ability of introduced organisms to successfully expand their distributions after invasion may have a genetic basis^9^, there must also be intraspecific differences (i.e., differences among haplotypes) in invasion success.

The Argentine ant, *Linepithema humile* Mayr, is a suitable invasive species for the exploration of intraspecific differences in traits between more- and less-successful haplotypes. *Linepithema humile*, native to South America, is one of the 100 most hazardous invasive species in the world^10^ and has been unintentionally introduced into almost all countries but not in Antarctica and oceanic islands^11,12^. In general, most ant species are multicolonial, with ant individuals being frequently hostile against non-nestmate conspecifics^13,14^. However, *L. humile* forms unique social structures called “supercolonies”, in which workers and reproductive castes (queens) move freely among interconnected nests within the same supercolony^15,16^, but show hostile behaviours towards individuals from different supercolonies, similar to the aggressive behaviour that occurs between colonies of multicolonial ants^17^. Moreover, recent studies based on a 1700-bp sequence of the mitochondrial COI-COII gene and a 524-bp sequence of Cyt b gene^18^ have shown that each *L. humile* supercolony has a single unique mitochondrial haplotype and functions as an independent reproductive unit^19^. As a result, the lack of hostility between *L. humile* workers within the same supercolony is maintained even at the transcontinental scale^20^. Importantly, the invasion success of *L. humile* is known to differ among supercolonies with different haplotypes^16,21^. In Europe, the United States (e.g., California), New Zealand, and Japan, a single *L. humile* supercolony having the same haplotype, “LH1”, extends its distribution range from tens to thousands of kilometres^16,18,20,22,23^ (Fig. 1). In contrast, invasions by other supercolonies with different haplotypes tend to be much less successful than LH1 in each introduced range^16,18,20,22,23^ (Fig. 1). However, the mechanisms and processes underlying the high invasion success of the LH1 supercolony have not been adequately explained. Although some hypotheses have been proposed to explain why the LH1 supercolony has been most successful (e.g., genetic drift resulting from bottlenecks^22^ and new selection pressures^16^), they do not consider trait variations among supercolonies (i.e., haplotypes). Previous studies have shown that the aggressiveness and insecticidal susceptibility of *L. humile* are significantly different among supercolonies^1,2,24^. Such trait variations of *L. humile* supercolonies can be expected to result in supercolony differences in invasion success; if the LH1 supercolony is superior with respect to some traits to other supercolonies, it may promote the dominance of LH1 in its introduced ranges.

**Figure 1:**
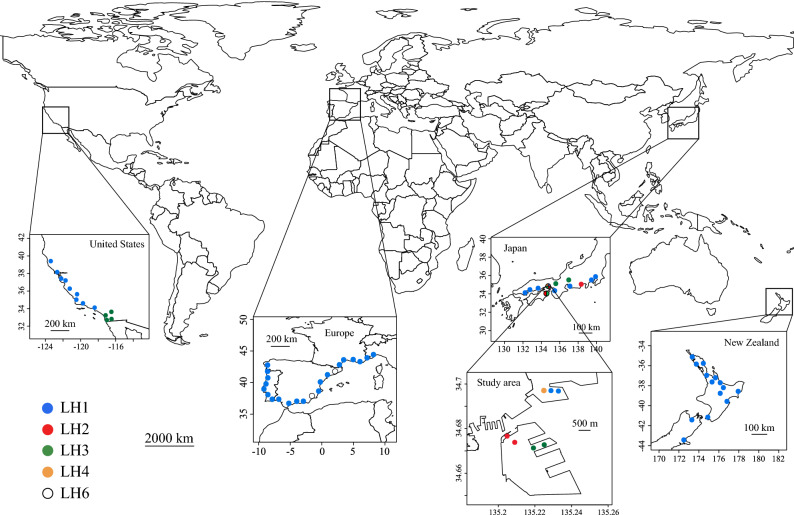
Distribution of Argentine ant (*Linepithema humile*) supercolonies of different haplotypes in each introduced region (partially modified by Tsutsui et al.^22^; Giraud et al.^16^; Corin et al.^23^; Inoue et al.^18^) and the locations of the sampling sites in this study. The name of each supercolony follows Inoue et al.^18^. The world map was plotted by R^25^ using 1: 50 m million scale vector data (ver. 4.0.0) provided by Natural Earth (https://www.naturalearthdata.com/downloads/50m-physical-vectors/).

To understand the mechanisms and processes underlying the high invasion success of the *L. humile* LH1 supercolony compared with other supercolonies, we focused on differences in trait levels among *L. humile* supercolonies. In particular, we investigated whether diet breadth differs among supercolonies. In general, diet breadth is an effective driver of invasion success^5^. Therefore, we hypothesized that the LH1 supercolony has greater diet breadth than other supercolonies, which results in the superior invasion success of LH1 and its subsequent dominance worldwide. Some researchers have attempted to describe the diet breadth of invasive species based on the range of the carbon (*δ*^13^C) and nitrogen (*δ*^15^N) stable isotope ratio^26,27^ and reported that the range of *δ*^13^C and *δ*^15^N in species with high invasiveness are broader than those of less-invasive species in various taxonomic groups^5^. In addition, diet breadth differs between more- and less-invasive species^5^. However, most of these studies are only “interspecific” comparisons between closely related species (i.e., same genus, same family). Thus, we applied this technique to measure the diet breadth of individual *L. humile* supercolonies (to identify intraspecific variation).

In Japan, four *L. humile* supercolonies with different mitochondrial haplotypes (LH1 (main supercolony), LH2, LH3, and LH4) have been found as of 2017^18,28–30^. The distribution ranges of the LH2, LH3, and LH4 supercolonies in Japan are narrower (representing less successful invasion) than the range of LH1^18,19^ (representing more successful invasion) (Fig. 1). Remarkably, all of the *L. humile* supercolonies have been detected in only Kobe City, Hyogo Prefecture, among the introduced prefectural regions and cities in Japan (see Sunamura et al.^28^ for details on the distribution of each *L. humile* supercolony). Furthermore, each of their ranges is limited to several square kilometres. Therefore, Kobe City (Japan) is one of the most suitable areas for a comparative study of the diet breadth of *L. humile* supercolonies.

Here, we investigated the ranges of *δ*^13^C and *δ*^15^N among the four *L. humile* supercolonies in Kobe Port, Kobe City, Japan, to measure diet breadth, which might be associated with the worldwide invasion success of LH1 (the main supercolony). Ranges of *δ*^13^C and *δ*^15^N were compared between the LH1 supercolony and the other supercolonies (LH2, LH3, LH4). If the working hypothesis is correct, LH1 should have broader *δ*^13^C and *δ*^15^N values than the other supercolonies. The findings of this research would lead to a better understanding of the process behind the invasion success of species introduced to new environments/habitats and their subsequent rapid population expansions.

## Results

**Figure 2:**
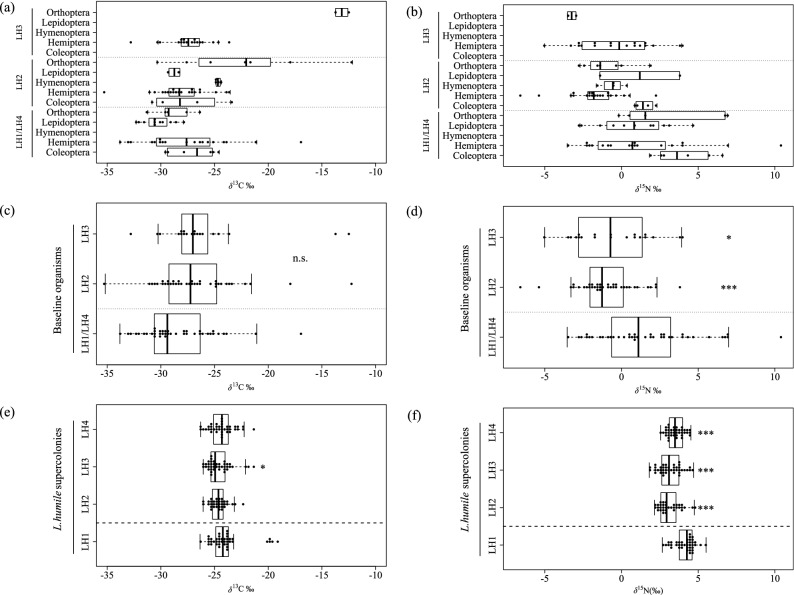
Comparisons of *δ*^13^C values of baseline organisms (arthropods) within the same sampling site of supercolonies LH1 and LH4 of *L. humile* and within sampling sites of other supercolonies (LH2, LH3) at the (**a**) taxonomic group and (**c**) community levels. The *δ*^15^N values of baseline organisms at taxonomic group and community levels within each *L. humile* sampling site are shown in (**b**) and (**d**), respectively. Comparisons of *δ*^13^C and *δ*^15^N among the four Japanese *L. humile* supercolonies are shown in (**e**) and (**f**), respectively. Differences in the *δ*^13^C and *δ*^15^N of baseline organisms at the community level among sampling sites (**c**, **d**) and of the four *L. humile* supercolonies (**e**, **f**) were analysed by Mann–Whitney *U* test. Asterisks (*p < 0.05, **p < 0.01, ***p < 0.001) denote significant differences.

**Figure 3:**
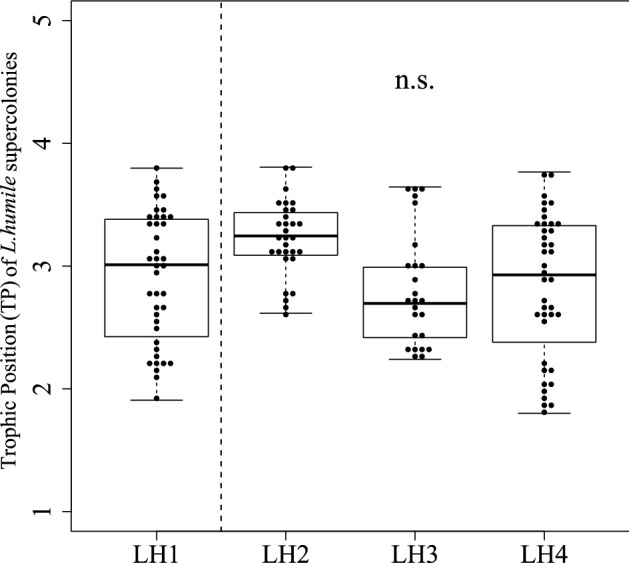
Differences in the trophic position (TP) of *L. humile* between the LH1 supercolony (main supercolony) and the other supercolonies (LH2, LH3, LH4) in Kobe, Hyogo, Japan, determined using Mann–Whitney *U* tests.

In total, 155 *L. humile* and 99 baseline organism (arthropod) samples from 2496 and 132 individuals, respectively, were obtained throughout the sampling period (Supplementary Tables S1 and S2). Although there were no clear differences in baseline *δ*^13^C among taxonomic groups sampled at the sites of LH1/LH4, lower *δ*^13^C values were found for Hemiptera than for other taxa within the sites of LH2 and LH3 (Fig. 2a). Additionally, there were no significant differences in the baseline *δ*^13^C of the arthropod community (i.e., whole arthropods) between sites LH1/LH4 and LH2 (Mann–Whitney *U* test, *W* = 542, *p* = 0.394) and/or LH3 (Mann–Whitney *U* test, *W* = 342, *p* = 0.128) (Fig. 2c). Clear differences in the baseline *δ*^15^N were not found among taxonomic groups within sampling sites, excluding the LH2 site, where Hemipterans had a lower *δ*^15^N value than other taxa, especially Coleopterans (Fig. 2b). The baseline *δ*^15^N of the arthropod community within the sampling site of LH1/LH4 was significantly higher than that of LH2 (Mann–Whitney *U* test, *W* = 1170, *p* < 0.001) and LH3 (Mann–Whitney *U* test, *W* = 576, *p* = 0.010) (Fig. 2d). Although haplotype LH3 had a statistically lower *δ*^13^C value than LH1 (Mann–Whitney *U* test, *W* = 1019, *p* = 0.037), differences in the *δ*^13^C of *L. humile* were not found between the LH1 supercolony and either the LH2 (Mann–Whitney *U* test, *W* = 860, *p* = 0.052) or LH4 supercolony (Mann–Whitney *U* test, *W* = 848, *p* = 0.510) (Fig. 2e). In contrast, the *δ*^15^N value of the LH1 supercolony was significantly higher than the corresponding values of the other supercolonies [versus LH2 (Mann–Whitney *U* test, *W* = 1158, *p* < 0.001), versus LH3 (Mann–Whitney *U* test, *W* = 1337, *p* < 0.001), versus LH4 (Mann–Whitney *U* test, *W* = 1208, *p* < 0.001)] (Fig. 2f). There were no significant differences in the trophic position (TP) of *L. humile* between LH1 and any of the other supercolonies [versus LH2 (Mann–Whitney *U* test, *W* = 396, *p* = 0.157), versus LH3 (Mann–Whitney *U* test, *W* = 542, *p* = 0.394), versus LH4 (Mann–Whitney *U* test, *W* = 868, *p* = 0.516)] (Fig. 3).

**Figure 4:**
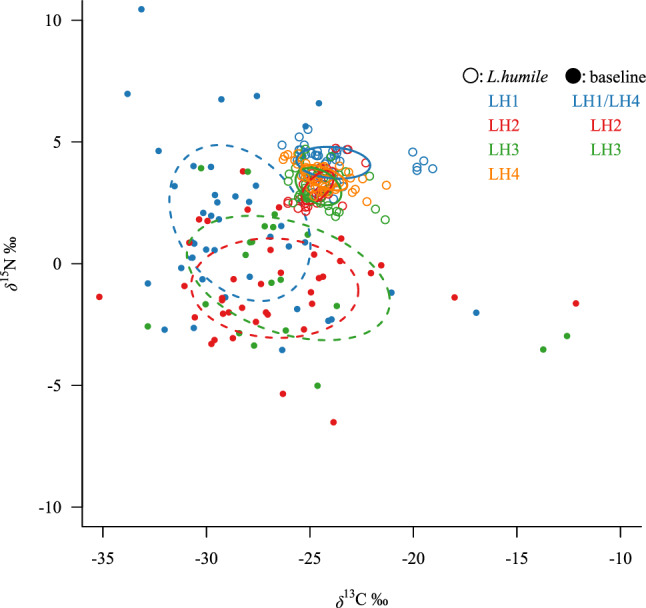
Differences in the standard ellipse areas (SEAs) among the four supercolonies of *L. humile* (*solid line ellipses and open dots*) established in Kobe, Hyogo, Japan, and among baseline organisms (arthropods) (*dashed line ellipses and filled dots*) collected within the sampling site of each supercolony (LH1/LH4, LH2, and LH3), calculated from the *δ*^13^C and *δ*^15^N biplot data using the R package “SIBER”^32^.

**Figure 5:**
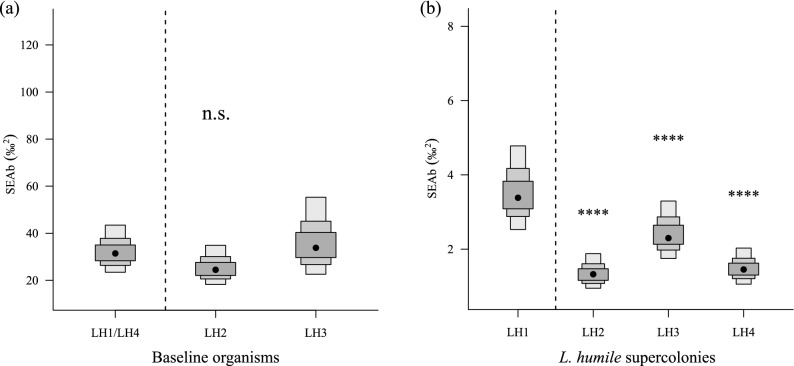
Comparisons of Bayesian standard ellipse area (●, SEAb: 10^5^ Bayesian iterations of SEA) among (**a**) baseline organisms collected within the sampling site of each supercolony (LH1/LH4, LH2, and LH3) and among (**b**) the four Japanese *L. humile* supercolonies, with 50%, 75%, and 95% credible intervals (CIs) shown, using the R package “SIBER”^32^. Asterisks indicate the significance of the pairwise differences between LH1 and the other supercolonies [*0.6 ≤ *P* < 0.69, **0.7 ≤ *P* < 0.79, ***0.8 ≤ *P* < 0.89, ****0.9 ≤ *P* ≤ 1; *P* is the likelihood of a difference between LH1 and the other supercolonies (or LH1/LH4 and the other sampling sites)]^32^.

**Figure 6:**
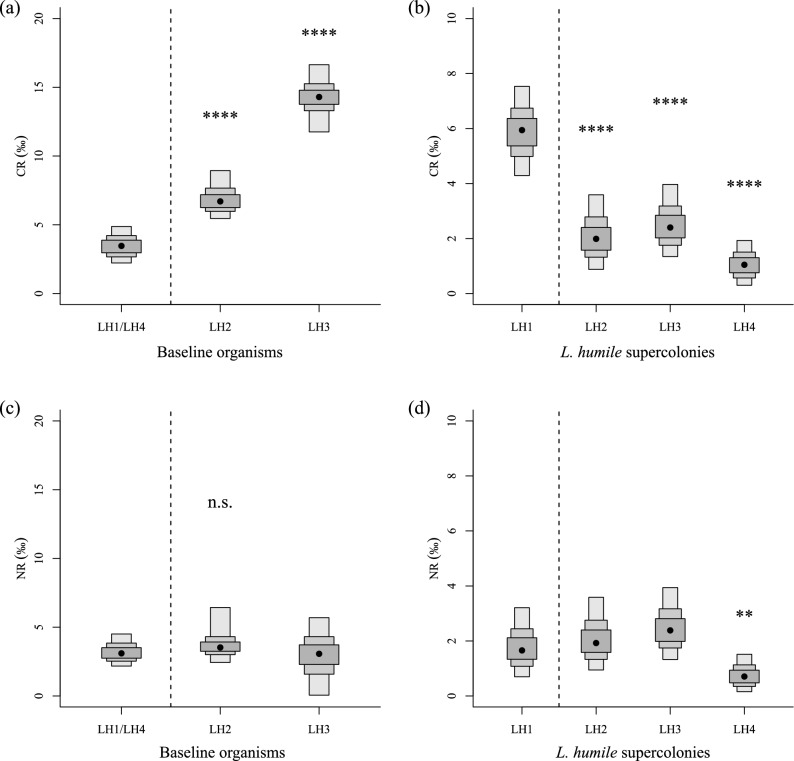
Differences in the range of *δ*^13^C (CR) among (**a**) baseline organisms collected within the sampling site of each supercolony (LH1/LH4, LH2, and LH3) and among (**b**) the four *L. humile* supercolonies, with 50%, 75%, and 95% credible intervals (CIs) shown, using the R package “SIBER”^32^. Differences in the range of *δ*^15^N (NR) among *L. humile* supercolonies and baseline organisms are shown in (**c**) and (**d**), respectively. Asterisks indicate the significance of the pairwise differences between LH1 and the other supercolonies [*0.6 ≤ *P* < 0.69, **0.7 ≤ *P* < 0.79, ***0.8 ≤ *P* < 0.89, ****0.9 ≤ *P* ≤ 1; *P* is the likelihood of a difference between LH1 and the other supercolonies (or LH1/LH4 and the other sampling sites)]^5^.

**Figure 7:**
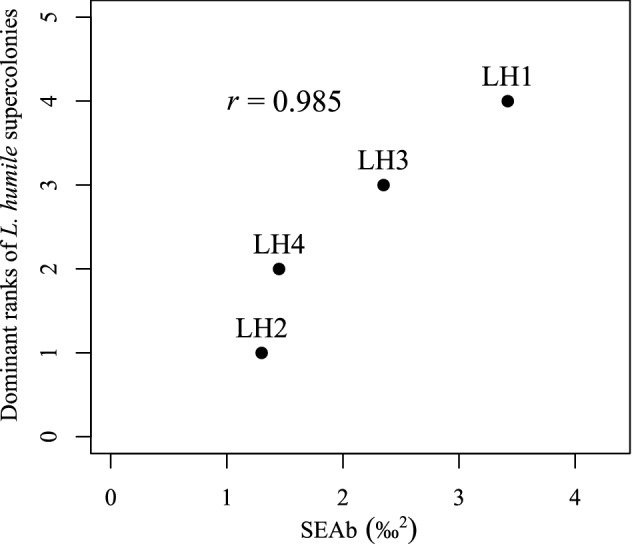
Relationship between the SEAb [10^5^ Bayesian iterations of SEAc (unbiased correction for differences in sample size of SEA)] values of the four supercolonies of *Linepithema humile* established in Kobe, Hyogo, Japan, and their dominance ranks in their introduced ranges. Dominance ranks were calculated using the number of introduced countries and regions in the world (see Inoue et al.^18^ for details on the distribution range of each *L. humile* supercolony).

Although there were no differences in the Bayesian-estimated standard ellipse areas (SEAb; an indicator of diet breadth) of baseline organisms between the sampling sites of LH1/LH4 and LH2 (Probability values^5^: *P* < 0.6) and/or LH3 (*P* < 0.6), the SEAb of *L. humile* LH1 was significantly larger than those of the other supercolonies (*P* > 0.9) (Figs. 4, 5). The breadth of SEAb increasing in the following order: LH1 > LH3 > LH4 ≥ LH2 (Fig. 5b). Additionally, the ratio of the SEAb of *L. humile* LH1 (10.87%) to the baseline SEAb of organisms collected at the LH1/LH4 sampling site tended to be higher than those of LH3 (6.77%), LH2 (5.28%), and LH4 supercolonies (4.58%) to each baseline SEAb within the sampling site of each supercolony (Fig. 4). The baseline *δ*^13^C range (CR: an indicator of the diversity of the baseline in the food web^31^) within the sites of LH2 (*P* > 0.9) and LH3 (*P* > 0.9) was significantly broader than that in the site of LH1/LH4 (Fig. 6a). The CR of *L. humile* LH1 was significantly broader than those of all the other supercolonies [versus LH2 (*P* > 0.9), versus LH3 (*P* > 0.9), versus LH4 (*P* > 0.9)] (Fig. 6b). There were no clear differences in the *δ*^15^N range (NR: an indicator of trophic diversity^31^) of baseline organisms between the sampling sites of LH1/LH4 and either LH2 (*P* < 0.6) or LH3 (*P* < 0.6) (Fig. 6c). Similarly, the NR values of *L. humile* did not significantly differ between LH1 and the other supercolonies except LH4 (*P* > 0.7) [versus LH2 (*P* < 0.6), versus LH3 (*P* < 0.6)] (Fig. 6d). The SEAb of the *L. humile* supercolonies showed a strong positive correlation with the dominance ranks of the supercolonies worldwide (*r* = 0.985, *P* = 0.051) reported by previous studies^12,16,19^ (Fig. 7).

## Discussion

Our results supported the hypothesis that the diet breadth of the LH1 supercolony is larger than those of the other supercolonies (LH2, LH3, LH4) and is associated with LH1′s superior invasion success.

The LH1 supercolony and baseline arthropods collected at the LH1/LH4 site had a higher *δ*^15^N than all other supercolonies (Fig. 2f,d). On the other hand, no differences in TP were found among the supercolonies (Fig. 3). These results suggest a higher baseline *δ*^15^N of arthropods within the LH1/LH4 sampling site (Fig. 2d), given that the *δ*^15^N (trophic levels) of *L. humile* differ little among supercolonies^33,34^. In addition, the *δ*^13^C of each *L. humile* supercolony overlapped with the baseline *δ*^13^C variations, whereas the *δ*^15^N of each supercolony was approximately 3–4% higher than the *δ*^15^N of the baseline organisms (Fig. 2). In general, although *δ*^15^N increases by approximately 3.4‰ with each increase in trophic level^33^, the carbon isotope ratio changes little increase (approximately 0–1%) with trophic level^31^, implying that the baseline organisms sampled in this study are likely available substrate for each *L. humile* supercolony.

The significantly broader CR of *L. humile* LH1 (Fig. 6b) is related to the high *δ*^13^C values of this supercolony (Figs. 2e, 4). The presence of C4 plants might explain the high *δ*^13^C values of consumers (i.e., *L. humile*) in our study area. In general, vascular plants are broadly classified into C3 or C4 plants according to their photosynthetic cycles, and they show characteristic *δ*^13^C distributions^35,36^. Specifically, C3 plants tend to exhibit a wide CR, from − 20 to − 35%, whereas the CR of C4 plants is narrower (− 10 to − 14%)^35,36^. Furthermore, since C4 plants usually have higher tolerance to drought, high temperatures, and human disturbance than C3 plants, they can more easily grow in areas such as port areas^37^. In fact, based on our field observations, C4 plants mostly representing *Poaceae* species were very dominant compared to C3 plants in sites paved with concrete of the study areas. Based on the above, the high *δ*^13^C for the LH1 supercolony is expected to be derived from C4 plants. Another potential cause of high *δ*^13^C in terrestrial organisms is corrosion^38^, which would be expected to lead to increases in *δ*^15^N and NR (an indicator of trophic diversity)^38,39^. However, in this study, the lack of clear, concurrent increases in *δ*^15^N and *δ*^13^C for *L. humile* (Fig. 2e) suggests that the high *δ*^13^C of the LH1 supercolony was not derived from a carrion diet. In light of the above, the members of the LH1 supercolony might be expected to have higher *δ*^13^C than those of the other supercolonies through the ingestion of C4 plant-derived resources, which would lead to the broader CR. The baseline *δ*^13^C of arthropods also indicates that LH1 supercolony can use the high-*δ*^13^C resource, which might be derived from C4 plant-based resources (Fig. 4).

Although the CR of baseline organisms within the sampling site of LH1/LH4 was significantly narrower than those of the others (Fig. 6a), the CR of *L. humile* LH1 showed opposite trend (Fig. 6b). These results suggest the possibility that *L. humile* LH1 consumed more diverse baseline resources than the other supercolonies, despite the relatively limited *δ*^13^C variation of baseline resources. In addition, such a broader range of CR in LH1 supercolony suggests that LH1 has a superior foraging (i.e., resource exploitation) ability^27,31^ to other supercolonies. A higher CR value implies the use of a diverse resource base^27,31^, Given that variations in *δ*^13^C occur in association with changes in the microenvironment, an increase in CR can lead to a greater range of resource exploration (i.e., more extensive resource acquisition). This is likely to reflect strong foraging ability, as mentioned by Jackson et al.^27^. Additionally, given that LH1 supercolony is more aggressive than other supercolonies^1,2^ and superior to other supercolonies in food acquisition due to its behavioural characteristics, the ability of workers to explore resources in a broad range of sites/environments might also be the greatest in LH1 supercolony. To validate this possibility, the behavioural characteristics of *L. humile* workers for food exploitation should be compared among supercolonies.

We found a clear intraspecific variation in the SEA of *L. humile*, which is an index of diet breadth, among the four supercolonies in Kobe, Hyogo, Japan (Fig. 4). Specifically, LH1 supercolony (the most successful supercolony) showed a significantly larger SEAb than the others (less-successful supercolonies) (Fig. 5b). On the other hand, there were no differences in the baseline SEAb of arthropods among the sampling sites (Fig. 5a). These results highlighted that the LH1 supercolony had more diet breadth than the other supercolonies when the diversities of the available baselines (arthropods) did not differ. Although the NR of *L. humile* did not differ among supercolonies other than LH4 (Fig. 6d), the CR of the LH1 supercolony was significantly broader than the CRs the other supercolonies (Fig. 6b), indicating that the larger SEAb for *L. humile* LH1 than for the other supercolonies (Fig. 5b) might be due to the broader CR of LH1. We thought that the broader CR of LH1 might be caused by its strong foraging resource exploitation (foraging) ability. Furthermore, the positive correlation between *L. humile* supercolony SEAb and the degree of their invasion success (i.e., distribution ranges in the world)^16,18,22^ (Fig. 7) support our hypothesis that an association between the diet breadth of the targeted species and its invasion success can be implied, even within species (between supercolonies). However, it should be noted that these results did not reveal a causal relationship between the dietary breadth of each *L. humile* supercolony and its invasion success. Monitoring future population dynamics of each *L. humile* supercolony with different diet breadth would be a promising approach to test the effects of diet breadth on invasion success. Additionally, while the present study showed that the diet breadth of *L. humile* differs among supercolonies, this result may have been due to the interaction between the different *L. humile* supercolonies and the surrounding environment. Although we attempted to minimize the effect of surrounding environment on the isotope ratios (i.e., diet breadth) by limiting sampling to the local scale (i.e., Kobe Port) and by performing baseline corrections of *δ*^15^N values, the possibility of a supercolony-environment interaction effect cannot be completely eliminated. To enable more robust comparisons among supercolonies, the effects of differences in landscape structures around sampling sites should be accounted for in future studies.

Previous studies on the mechanisms and processes of biological invasions have mainly emphasized interspecific differences in biological traits and invasion success rather than intraspecific differences^7,8^. For example, even indigenous species that are not dominant in their native ranges can easily become species with invasiveness when invading new environments/areas, which may be due to their flexible diet changes^40^. In this context, our study highlights the importance of trait–invasion success relationships, especially at the within-species level rather than at the among-species level, which are well known. Therefore, focusing on the intraspecific variation in addition to the interspecific variation in trait levels should be helpful for assessing potential biological invaders that successfully invade new habitats and then become dominant.

## Materials and methods

### Sampling of *L. humile* and other arthropods

This study was performed at Kobe Port (34°41′–34°40′ N and 135°13′–135°12′ E) in Kobe City, Hyogo Prefecture, Japan, on sunny days from August 2017 to April 2018. As mentioned above, four *L. humile* supercolonies with different haplotypes (LH1, LH2, LH3, LH4) had previously been detected adjacent to Kobe Port^18,28^, all of which structured their nests mainly in roadside cracks adjacent to parks, street trees, and factory premises. We collected 5–10 *L. humile* samples consisting of 10–20 workers of each supercolony per sample and samples for baseline organisms (i.e., primary consumers, including aphids, mealybugs, and grasshoppers) in each sampling site once a season (see Fig. 3, Supplementary Table S1). Baseline organisms were used to estimate the TP of each *L. humile* supercolony (see *Stable isotope analysis*). However, when calculating the TP of LH4, we used the same baseline *δ*^13^C and *δ*^15^N as for LH1 because LH4 and LH1 were found in very close proximity (ca. 150 m) to each other. Otherwise, the distance between sampling sites was at least 200 m. *L. humile* workers and other arthropods were sampled by sucking with a fluke tube and using a 68 × 68 cm beating net (N-type, Mushi-sha Ltd., Tokyo).

### Stable isotope analysis

All samples collected were immediately frozen (− 40 °C) and then oven dried at 60 °C for 24 h. After drying, we removed the gaster or abdomen of each *L. humile* individual and other arthropods as much as possible because it can contain residual food particles and thus potentially affect the detected isotopic signature^41,42^. In addition, some samples that contained lipids were excluded before stable isotope analysis because their *δ*^13^C values were lower than those of muscle^43^. Then, analytical samples were homogeneously crushed using an agate mortar and pestle. These samples and in-house standards [i.e., L-alanine (*δ*^13^C: − 19.0‰, *δ*^15^N: 22.7‰), glycine (*δ*^13^C: − 34.9‰, *δ*^15^N: 2.2‰), and L-threonine (*δ*^13^C: − 9.45‰, *δ*^15^N: − 2.9‰)]^44^ were weighed to approximately 0.5 mg (approximately 6–7 individuals per analytical sample) into 5 × 8 mm tin cups and then analysed with a Delta V isotope mass spectrometer (Thermo Electron Corporation, Waltham, Massachusetts, USA) equipped with an elemental analyser. If a sample of a given species weighed less than 0.5 mg, other individuals of this species were added until a total of 0.5 mg was reached.

The relative abundances of carbon (*δ*^13^C) and nitrogen (*δ*^15^N) stable isotopes within each sample are expressed in delta notation and were calculated using the following equation:

$$\delta R(\permil) = \left( {\frac{{R_{sample} }}{{R_{standard} }} - 1} \right) \times 1000$$

where *R* is the ratio of ^13^C/^12^C and ^15^N/^14^N for samples and the standard reference materials Pee Dee belemnite (for CO_2_) and atmospheric nitrogen (for N_2_).

TP was corrected as is common practice with the following equation:

$${\text{TP}} = 2 + \frac{{\left( {\delta^{15} {\text{N}}_{ants} - \delta^{15} {\text{N}}_{base} } \right)}}{3.4}$$

where 2 is the baseline TP^33^, *δ*^15^N_ant_ is the *L. humile* isotope value, *δ*^15^N_base_ is the baseline mean *δ*^15^N, and 3.4 represents the fractionation between trophic levels^33^.

### Statistical analysis

All statistical analyses were conducted with the free statistical software R ver. 3.6.1^25^. We compared *δ*^13^C, *δ*^15^N, and TP between *L. humile* LH1 and each of the other supercolonies. Since the distributions of *δ*^13^C, *δ*^15^N, and TP were significantly different from a normal distribution (Shapiro–Wilk test, *p* < 0.05) and showed unequal variance among the four *L. humile* supercolonies (Levene's test, *p* < 0.05), differences in the isotopic ratio among supercolonies were assessed by Mann–Whitney *U* test (*p* < 0.05).

To compare CR and NR among the four *L. humile* supercolonies, SEAs were calculated from the *δ*^13^C and *δ*^15^N data. The SEA is a measure of variability in *δ*^13^C and *δ*^15^N and represents approximately 40% of the spread of these data, which is expected to express the core range of *δ*^13^C and *δ*^15^N^32^. This analysis was performed using the package “SIBER” (Stable Isotope Bayesian Ellipses) in R^32^. The following equation was used to correct the SEAs for the use of *δ*^13^C and *δ*^15^N bivariate data^32^:

$${\text{SEAc}} = {\text{SEA}} \times \frac{n - 1}{{n - 2}}$$

The SEAc correction (SEAc: unbiased correction for differences in sample sizes of SEAs) accounts for loss of a second degree of freedom. In addition, a Bayesian-estimated SEAc (SEAb: 10^5^ Bayesian iterations of SEAc), its credible intervals (10^5^ posterior draws), CR (maximum *δ*^13^C–minimum *δ*^13^C), and NR (maximum *δ*^15^N–minimum *δ*^15^N) from multiple Bayesian iterations (10^5^) were calculated by a bootstrapping procedure, which allowed a robust comparison among the four *L. humile* supercolonies that had different sample sizes. Probability (*P*) values ranging from zero to one were calculated as the likelihood of the differences in SEAb, CR and NR; zero indicated no difference, and *P* > 0.6 was considered significant^5^. Then, we calculated Spearman's rank correlation coefficients to confirm the relationship between SEAb and dominance rank (number of introduced countries in the world) as an indicator of the degree of invasion success for each supercolony. The number of introduced countries was determined from Inoue et al.^18^.

A larger SEA indicates greater diet breadth, as reported by previous studies^5,45^. A larger CR is expected in food webs containing multiple basal resources with varying *δ*^13^C values, implying a broad total range of exploited resources^27,31^. On the other hand, a larger NR suggests that the focal organism belongs to various trophic levels and thus shows a high degree of trophic diversity^31^. These analyses were conducted on baseline organisms (arthropods) collected at sampling sites of each *L. humile* supercolony in the same way as on *L. humile*.

## Supplementary Information

Supplementary Information.
