# Supplementary material for: Intraspecific differences in the invasion success of the Argentine ant Linepithema humile Mayr are associated with diet breadth
Source: Sci Rep. 2021 Feb 3;11:2874. doi: 10.1038/s41598-021-82464-1 (PMC7859199; doi:10.1038/s41598-021-82464-1)
Supplement: Supplementary file 1 — Supplementary Information. [file 41598_2021_82464_MOESM1_ESM.pdf]

Intraspecific differences in the invasion success of the Argentine ant *Linepithema humile* Mayr are associated with diet breadth

Yugo Seko, Koya Hashimoto, Keisuke Koba, Daisuke Hayasaka, Takuo Sawahata

Supplementary table S1. Isotopic measurement results for the baseline organisms (arthropods) collected at the sampling site of each *Linepithema humile* supercolonies (LH1, LH2, LH3, LH4).

Sampling site	Order	Species	δ^{13}C value	δ^{15}N value
LH1, 4	Hemiptera	*Aphidoidea spp.*	-29.77872988	3.977365263
LH1, 4	Hemiptera	*Aphis gossypii*	-33.8100193	6.977928215
LH1, 4	Hemiptera	*Cicadellidae spp.*	-26.91054973	1.105880134
LH1, 4	Hemiptera	*Cimicomorpha*	-30.62200981	4.011124078
LH1, 4	Coleoptera	*Elateridae spp.*	-25.20856402	5.64544791
LH1, 4	Hemiptera	*Eysarcoris ventralis*	-16.96396012	-2.011845766
LH1, 4	Lepidoptera	*Oraesia emarginata*	-32.30959806	4.633676352
LH1, 4	Lepidoptera	*Oraesia emarginata*	-31.53727705	3.187011818
LH1, 4	Hemiptera	*Aphidoidea spp.*	-33.13950892	10.45210749
LH1, 4	Orthoptera	*Atractomorpha lata*	-29.27687549	6.753531383
LH1, 4	Coleoptera	*Chrysolina aurichalcea*	-29.58744931	2.822615192
LH1, 4	Coleoptera	*Chrysolina aurichalcea*	-29.39616874	1.82275851
LH1, 4	Hemiptera	*Cimicomorpha*	-30.01320284	0.580632682
LH1, 4	Coleoptera	*Elateridae spp.*	-25.31860177	4.413251145
LH1, 4	Orthoptera	*Meconematidae sp.*	-27.56049231	6.889559551
LH1, 4	Hemiptera	*Uroleucon nigrotuberculatum*	-30.69811068	0.886447834
LH1, 4	Orthoptera	*Atractomorpha lata*	-29.60604202	0.557833463
LH1, 4	Orthoptera	*Atractomorpha lata*	-31.2151792	-0.174981937
LH1, 4	Hemiptera	*Cicadellidae spp.*	-29.47186443	2.521263086
LH1, 4	Hemiptera	*Eysarcoris ventralis*	-21.06141426	-1.187437313
LH1, 4	Orthoptera	*Phaneroptera falcata*	-26.38379202	1.550456217
LH1, 4	Hemiptera	*Sogatella spp.*	-27.60828678	3.20449693
LH1, 4	Lepidoptera	*Sphingidae sp.*	-27.90745311	-0.535935666
LH1, 4	Lepidoptera	*Sphingidae sp.*	-28.59821922	2.766195974
LH1, 4	Hemiptera	*Aphidoidea spp.*	-30.19010273	-0.639611443
LH1, 4	Hemiptera	*Aphidoidea spp.*	-32.82847141	-0.810989133
LH1, 4	Hemiptera	*Nezara antennata*	-26.33890288	-3.542291476
LH1, 4	Coleoptera	*Chrysolina aurichalcea*	-27.93677871	2.540823857
LH1, 4	Hemiptera	*Nezara antennata*	-25.23599319	0.878956851
LH1, 4	Hemiptera	*Nezara antennata*	-26.01779953	0.710779891
LH1, 4	Lepidoptera	*Oraesia emarginata*	-30.67660449	0.249039597
LH1, 4	Lepidoptera	*Oraesia emarginata*	-30.58551054	0.826214964
LH1, 4	Coleoptera	*Elateridae spp.*	-24.56530146	6.591997859
LH1, 4	Lepidoptera	*Geometridae spp.*	-29.08796601	-1.380983251
LH1, 4	Hemiptera	*Nezara antennata*	-23.95467167	-2.28754663
LH1, 4	Hemiptera	*Nezara antennata*	-25.61638552	-1.861631014
LH1, 4	Hemiptera	*Nezara antennata*	-24.1098317	-2.344269037

LH1, 4	Lepidoptera	*Geometridae spp.*	-30.60953532	-2.63783237
LH1, 4	Lepidoptera	*Geometridae spp.*	-29.7696691	1.96961927
LH1, 4	Lepidoptera	*Oraesia emarginata*	-30.1520635	2.088039733
LH1, 4	Lepidoptera	*Oraesia emarginata*	-32.01898899	-2.708486596
LH2	Hemiptera	*Aphidoidea spp.*	-28.68631023	-0.635551044
LH2	Hemiptera	*Aphis gossypii*	-28.91667245	-1.99847975
LH2	Hymenoptera	*Apidae sp.*	-24.79689096	0.37773098
LH2	Hymenoptera	*Apidae sp.*	-24.3670186	-0.53154849
LH2	Coleoptera	*Elateridae spp.*	-26.49375553	2.315149983
LH2	Hemiptera	*Pseudococcidae spp.*	-29.75687752	-3.29306392
LH2	Hemiptera	*Pseudococcidae spp.*	-29.60981592	-3.133593337
LH2	Hemiptera	*Uroleucon nigrotuberculatum*	-28.00859281	2.223033436
LH2	Hemiptera	*Aphidoidea spp.*	-31.06089223	-0.914872189
LH2	Hemiptera	*Aphidoidea spp.*	-30.56006008	-2.202522855
LH2	Hymenoptera	*Apidae sp.*	-24.89561763	-1.644871067
LH2	Hymenoptera	*Apidae sp.*	-24.56344353	-0.591969022
LH2	Orthoptera	*Acrida cinerea*	-12.15133486	-1.630129066
LH2	Orthoptera	*Atractomorpha lata*	-18.01217796	-1.384027543
LH2	Hemiptera	*Cryptotympana facialis*	-28.27640053	-1.80875114
LH2	Hemiptera	*Erthesina fullo*	-23.54022101	0.111599458
LH2	Hemiptera	*Graptopsaltria nigrofuscata*	-24.95062448	-1.172658089
LH2	Hemiptera	*Graptopsaltria nigrofuscata*	-26.90290601	0.566922441
LH2	Lepidoptera	*Papilio xuthus*	-28.23341635	3.794027907
LH2	Orthoptera	*Phaneroptera falcata*	-21.55387455	-0.065201231
LH2	Orthoptera	*Phaneroptera falcata*	-22.05169134	-0.384736274
LH2	Coleoptera	*Scarabaeidae sp.*	-23.48816269	1.035309212
LH2	Orthoptera	*Atractomorpha lata*	-30.35525149	1.820072826
LH2	Coleoptera	*Chrysolina aurichalcea*	-30.82481211	0.864315502
LH2	Orthoptera	*Ectatoderus annulipedus*	-25.28740816	-2.698684284
LH2	Hemiptera	*Pseudococcidae spp.*	-29.24155003	-1.498777114
LH2	Coleoptera	*Chrysolina aurichalcea*	-29.94689429	1.757546646
LH2	Orthoptera	*Ectatoderus annulipedus*	-27.60812121	-2.3900233
LH2	Hemiptera	*Erthesina fullo*	-26.40411963	-0.369335956
LH2	Hemiptera	*Leptocorisa chinensis*	-29.19639997	-2.053572902
LH2	Hemiptera	*Nezara antennata*	-26.30378616	-5.344633429
LH2	Hemiptera	*Nezara antennata*	-27.03923046	-2.086324711
LH2	Hemiptera	*Nezara antennata*	-23.85966295	-6.511788791
LH2	Lepidoptera	*Oraesia emarginata*	-29.24054669	-1.409454
LH2	Hemiptera	*Pseudococcidae spp.*	-35.17160787	-1.362485021
LH2	Hemiptera	*Pseudococcidae spp.*	-27.12342649	-1.99993089
LH2	Hemiptera	*Pseudococcidae spp.*	-27.3630414	-0.831280131
LH2	Hemiptera	*Psyllidae spp.*	-28.72257319	-3.05737614

LH3	Hemiptera	*Aphidoidea spp.*	-30.25668126	3.924713317
LH3	Hemiptera	*Psyllidae spp.*	-30.03763147	-1.663690576
LH3	Hemiptera	*Psyllidae spp.*	-27.79468503	0.896753249
LH3	Orthoptera	*Acrida cinerea*	-13.72845212	-3.523646734
LH3	Hemiptera	*Aphis nerii*	-28.41276311	-2.85221619
LH3	Hemiptera	*Erthesina fullo*	-25.10538731	1.195100086
LH3	Orthoptera	*Locusta migratoria*	-12.58091404	-2.969543994
LH3	Hemiptera	*Coreidae sp.*	-26.85652645	-0.782711473
LH3	Hemiptera	*Erthesina fullo*	-26.70239439	2.027240144
LH3	Hemiptera	*Erthesina fullo*	-27.89070289	0.881568642
LH3	Hemiptera	*Erthesina fullo*	-27.18467859	1.539195915
LH3	Hemiptera	*Erthesina fullo*	-26.77399122	1.504687738
LH3	Hemiptera	*Aphidoidea spp.*	-32.83148141	-2.573631924
LH3	Hemiptera	*Alcimocoris japonensis*	-28.10477187	0.362121103
LH3	Hemiptera	*Nezara antennata*	-26.16532598	-2.742353363
LH3	Hemiptera	*Nezara antennata*	-27.69842133	-3.36066781
LH3	Hemiptera	*Nezara antennata*	-24.63223063	-5.011160469
LH3	Hemiptera	*Nezara antennata*	-26.41075211	-0.657521522
LH3	Hemiptera	*Nezara antennata*	-23.70197244	-1.735817804
LH3	Hemiptera	*Psyllidae spp.*	-28.0006433	3.781762714

Supplementary table S2. Isotopic measurement results for the *Linepithema humile* supercolonies in Japan.

Supercolony	δ^{13}C value	δ^{15}N value
LH1	-23.5505637	4.212430443
LH1	-23.90132413	4.62002483
LH1	-24.92430347	4.538926232
LH1	-23.85858063	2.661124247
LH1	-25.20386983	4.470552439
LH1	-24.40680829	3.260633588
LH1	-23.2201383	4.687564911
LH1	-25.09481912	5.516968743
LH1	-25.27227437	4.878813345
LH1	-24.85490756	2.930655719
LH1	-24.53892051	3.589662971
LH1	-25.31550153	3.630354612
LH1	-24.49678046	3.019979997
LH1	-23.71142079	3.323991123
LH1	-23.82253539	3.753887259
LH1	-19.49913911	4.226573982
LH1	-19.06869516	3.90514701
LH1	-19.82847878	3.965849937
LH1	-20.03268939	4.588801282
LH1	-19.82647671	3.832502524
LH1	-24.58932624	4.44251718
LH1	-24.63937786	4.61865518
LH1	-24.66140058	4.4365464
LH1	-23.39209144	3.172731372
LH1	-24.0047233	4.372858084
LH1	-23.70641563	4.537054525
LH1	-23.68239085	4.671397067
LH1	-23.82453746	4.455453869
LH1	-23.93865516	3.783741157
LH1	-23.74645693	4.27334509
LH1	-24.1863442	4.204740814
LH1	-24.52365617	3.938641916
LH1	-24.01151788	3.127437438
LH1	-24.73858972	4.463889368
LH1	-24.27684254	4.102471461
LH1	-26.34699192	4.875945498
LH1	-25.43892341	4.679350043
LH1	-25.52325141	4.679350043

LH1	-25.61169296	4.249421599
LH1	-25.51708107	5.209562028
LH2	-24.4370127	2.773393046
LH2	-24.4370127	2.773393046
LH2	-25.19792161	2.155752737
LH2	-25.09779323	2.342046548
LH2	-25.21675764	2.484739681
LH2	-24.61617728	3.551948767
LH2	-24.79075751	3.480490276
LH2	-24.98339776	3.35047552
LH2	-24.46668042	3.899316434
LH2	-24.97436775	3.434836239
LH2	-25.22118808	2.952491421
LH2	-25.20714139	2.325244662
LH2	-23.42722572	2.373876136
LH2	-24.90313099	2.773845193
LH2	-26.05696584	2.564432113
LH2	-23.75442034	4.744881862
LH2	-24.11230011	3.52261416
LH2	-23.15898243	4.692257826
LH2	-22.30233343	4.145166434
LH2	-23.92616149	3.79367759
LH2	-24.53394008	2.978501487
LH2	-24.43110106	2.738218153
LH2	-24.6563185	2.490984475
LH2	-24.13698147	3.074813402
LH2	-24.68922699	2.874246322
LH2	-25.57333505	2.273548962
LH2	-24.43489319	2.932231803
LH2	-24.1058043	2.862896767
LH2	-26.06491158	2.48254457
LH2	-25.48592082	2.652910658
LH2	-24.20144576	3.921741815
LH2	-24.89870284	3.6998697
LH2	-24.48219971	3.934618322
LH2	-25.32754679	3.264049475
LH3	-24.06112616	2.154527241
LH3	-23.71338086	2.152534114
LH3	-23.76043499	1.941713038
LH3	-21.36231077	1.808929151
LH3	-24.12327644	2.386638152
LH3	-21.83394817	2.2452145

LH3	-24.34354358	3.504870504
LH3	-25.21973175	3.264597286
LH3	-24.47025597	2.705715851
LH3	-23.58892797	2.119088529
LH3	-25.49610177	2.832401456
LH3	-25.17904802	2.71429645
LH3	-25.25028478	2.844311205
LH3	-24.97938442	2.554507323
LH3	-25.30446485	2.902867469
LH3	-25.418845	4.369751504
LH3	-22.11887733	3.598595282
LH3	-25.69576537	3.938023117
LH3	-25.49509844	4.184157921
LH3	-25.21516807	4.708186859
LH3	-24.62958036	3.698358582
LH3	-23.27210535	3.780769808
LH3	-24.49074769	2.488005756
LH3	-23.90662208	3.345876832
LH3	-24.15754928	2.977508581
LH3	-24.91650121	3.832400937
LH3	-25.12629281	3.497791502
LH3	-25.26409709	3.796656309
LH3	-23.97655261	4.403322082
LH3	-24.85685459	3.818500249
LH3	-25.06427568	2.703426184
LH3	-25.5537954	2.731160199
LH3	-25.24321776	3.103588392
LH3	-25.8150097	2.50235458
LH3	-25.37279651	2.754932211
LH3	-24.07803743	3.087740383
LH3	-23.58954611	3.648363674
LH3	-26.04845713	3.387862039
LH3	-25.53425575	4.524956627
LH3	-25.9990938	3.687983694
LH4	-24.09493939	2.950534781
LH4	-23.57703153	4.017699929
LH4	-24.29676621	3.136768022
LH4	-23.69203046	3.150640965
LH4	-24.30886066	3.016029541
LH4	-23.566134	3.331595205
LH4	-24.23288862	3.478462243
LH4	-24.74969843	3.12617982

LH4	-24.64373742	3.999443292
LH4	-23.87002216	3.292893756
LH4	-25.22319474	3.547978851
LH4	-24.99945112	3.11029559
LH4	-23.84862627	3.614474947
LH4	-22.95064176	2.930656882
LH4	-24.25096347	3.751437056
LH4	-24.61015727	3.438806155
LH4	-26.06599585	4.483886594
LH4	-26.22552606	4.418382977
LH4	-22.2452975	2.551529886
LH4	-23.75631948	3.054716763
LH4	-22.45112095	3.490177824
LH4	-25.73050322	4.249461971
LH4	-25.58835661	4.522127575
LH4	-26.29108138	4.282301259
LH4	-21.30593985	3.224478129
LH4	-25.18894467	3.954903508
LH4	-23.12982095	3.763838558
LH4	-23.04072906	3.519036592
LH4	-25.19695293	4.121090208
LH4	-22.8104916	3.056301168
LH4	-24.29638195	2.913962575
LH4	-24.80852025	2.766019531
LH4	-24.39716418	2.916941294
LH4	-24.69096548	3.555256625
LH4	-25.61138596	4.095079405
LH4	-24.39787069	3.963342837
LH4	-24.69610749	3.367061528
LH4	-25.40981901	3.612705655
LH4	-24.30839965	4.000981856
